# Endemics and Cosmopolitans: Application of Statistical Mechanics to the Dry Forests of Mexico

**DOI:** 10.3390/e21060616

**Published:** 2019-06-22

**Authors:** Michael G. Bowler, Colleen K. Kelly

**Affiliations:** 1 Department of Physics, University of Oxford, Keble Road, Oxford OX1 3RH, UK; 2 Department of Integrative Biology, University of South Florida, Tampa, FL 33620, USA

**Keywords:** statistical mechanics, resource partitioning, distribution of species, seasonally dry tropical forest, biotic resistance

## Abstract

Data on the seasonally dry tropical forests of Mexico have been examined in the light of statistical mechanics. The results suggest a division into two classes of species. There are drifting populations of a cosmopolitan class capable of existing in most dry forest sites; these have a statistical distribution previously only observed (globally) for populations of alien species. We infer that a high proportion of species found only at a single site are specialists, endemics, and that these prefer sites comparatively low in species richness.

## 1. Introduction

The techniques of statistical physics have been applied to problems of community structure with some success, primarily in achieving an understanding of the principles underlying the rather universal form of the species abundance distribution. This is traced to the rate of change of a population being proportional to the number of individuals each species contains [1,2,3,4]. A rather more surprising application is to the distribution of alien species over the globe, where it was found that the number of species at *n* sites to which they are alien is exponentially distributed with *n.* At the same time, the distribution of the number of sites as a function of the number of species present is consistent with being drawn from an underlying exponential probability distribution. Beyond that, the number of the pairs of sites sharing *p* species is exponentially distributed with *p* [5]. The relationship between these various exponential probability distributions was elucidated in [6]. In [5] it is speculated that similar principles may apply more generally to community assembly, for there are examples of the number of species with the number of sites occupied being distributed exponentially in populations of heteroflagellates and of tree species in the seasonally dry tropical forests of Mexico. Having clarified in [6] the roles of the alien species’ exponentials, we have returned to the dry forests of Mexico, where the data [7] contain not only the exponential distribution of the number of species with the number of sites (which first drew our attention), but also list the number of species at each site, and beyond that the number of species common to each pair of sites. These three aspects of the data set are explained by a category of cosmopolitan species, distributed in accord with the model for alien species, and in addition, a category of specialists, endemic species found preferentially at high rank sites, i.e., those that are relatively species poor.

## 2. The Underlying Model

An exponential distribution of the number of species with the number of sites at which the species are found can be described using simple statistical mechanics. 

A given resource is to be divided into a given number of pieces, the cuts to be made at random. The most probable configuration is an exponential distribution of the number of pieces with their length. In ecology, this is known as MacArthur’s broken stick [8], in the statistical mechanics of gases, a microcanonical ensemble. It is also the maximum entropy solution for a uniform prior. If the same resource is also divided between the different sites in the same sort of way, the distribution of the number of sites with the number of species each site contains is, for our particular application, drawn from an underlying exponential distribution. These exponential distributions are themselves sufficient to generate the exponential distribution of the number of pairs (and higher multiplets) of sites with the number of species shared, without any further assumptions [6]. It is worth remarking that the placing of the cuts could be accomplished statically (as in MacArthur’s broken stick) or dynamically, with species accepted and rejected from sites, and sites growing and contracting in receptivity; described by the appropriate master equations. The original application of these ideas was to the statistical mechanics governing the global distribution of alien species, but the data of [7] invite their application to the structure of the seasonally dry tropical forests of Mexico.

## 3. Relevant Aspects of the Data

**Figure 1:**
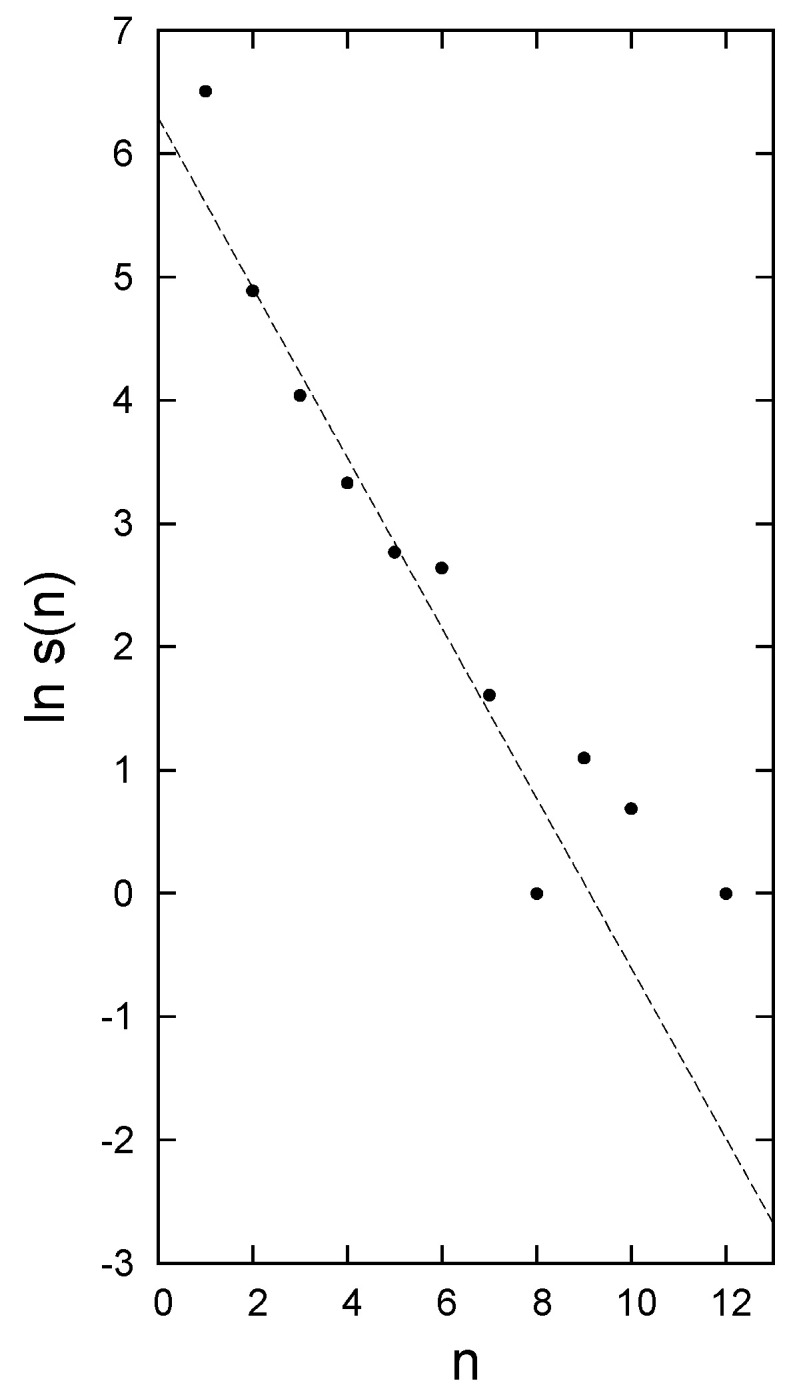
The natural logarithm of the number of species found at *n* sites in [7] is plotted against *n*. For *n >* 1 the distribution is consistent with an exponential, s(n)=540exp(−0.69n). Note that the statistics become erratic beyond n ~7. For *n* = 1 there is an excess of ~400 above the extrapolated exponential. We argue that these must be largely *endemics.*

The data presented in [7] comprise 917 species from 20 different widely scattered sites in Mexico. Of these, 670 are found at one site only, and no species are found at more than 12 sites. The richest site contains 124 species. The distribution of the number of species *s*(*n*) found at *n* sites agrees well with an exponential for *n* > 1 (Figure 5 of [7], Figure 1 of the present paper), and the number of pairs sharing *p* species in common is consistent with an exponential distribution in *p* (see Figure 3, upper panel).

**Figure 2:**
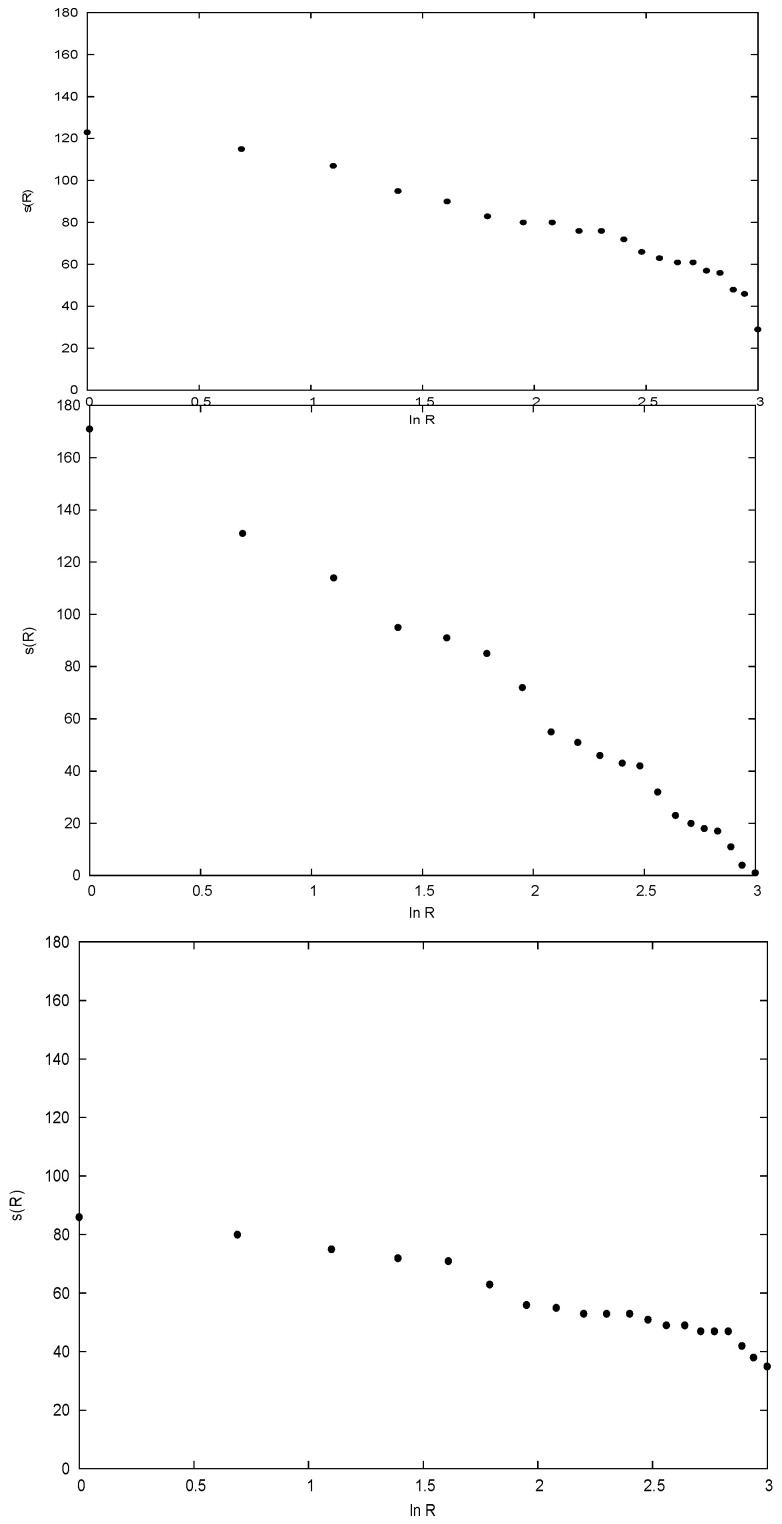
The number of species s(R) at sites of rank *R* plotted against lnR. **Upper panel:** The data in [7], where the highest rank sites have occupancy ~40 species. **Middle panel:** The distribution for an underlying exponential for *cosmopolitan* species; the highest rank sites would have negligible population. **Lower panel:** The distribution, assuming that sites have receptivity for cosmopolitans similar to the data.

However, the species richness of the individual sites, Figure 2 upper panel, is not consistent with having been drawn from an underlying exponential probability. The most probable receptivity (or species richness) of a site of rank *R* is proportional to $lnR_{0}-lnR$, where the richest site is rank 1, and for a simple exponential, $R_{0}$ is the number of sites +1, see [6]. For 20 sites and a pure exponential, $lnR_{0}=3.04$. In Figure 2 (upper panel) we plot the number of species s(R) against lnR for the data. It is a reasonable approximation to a straight line, but extrapolates to zero species at lnR≈4.8. 

**Figure 3:**
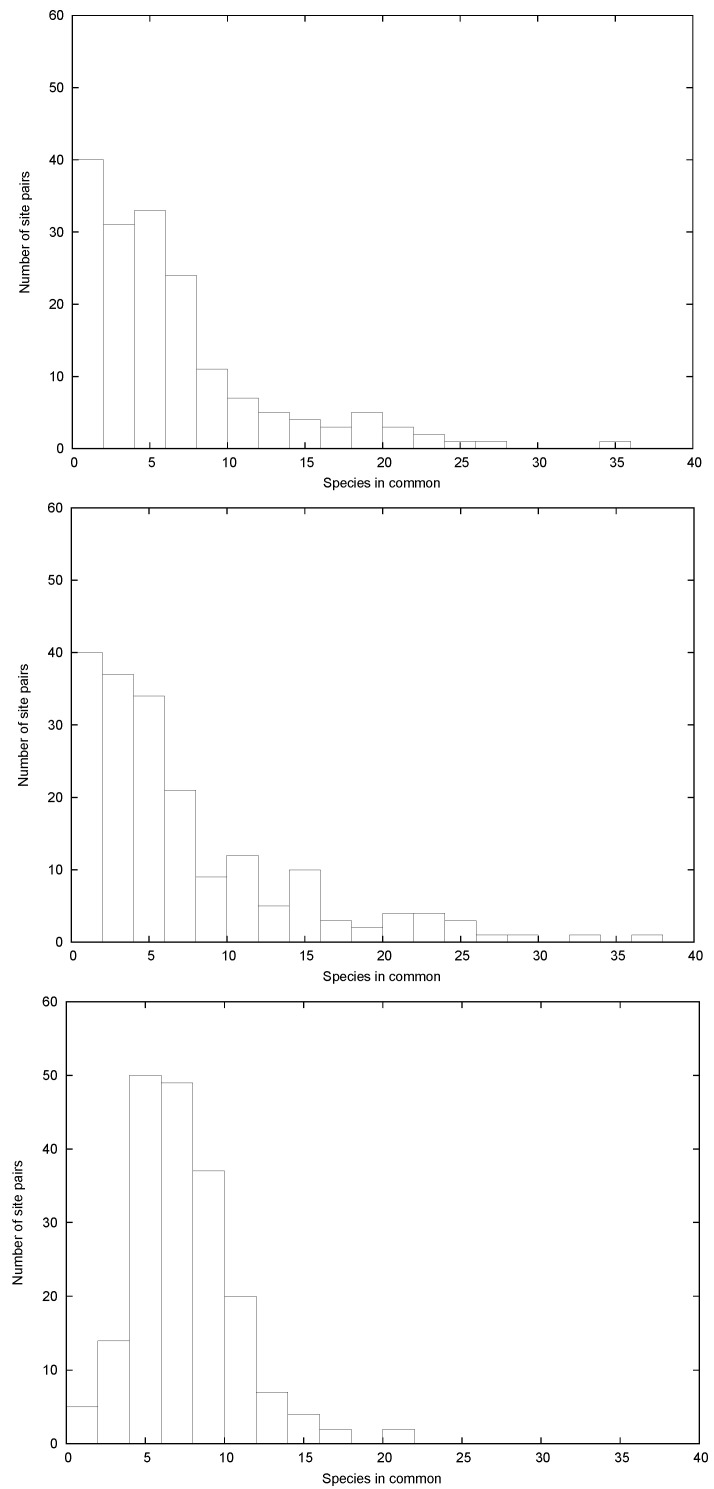
The number of pairs of sites as a function of the number of species in common. Only species found at two or more sites contribute. **Upper panel:** The data of [7]. **Middle panel:** The model distribution for the cosmopolitan receptivities determined by a simple exponential underlying probability; *cf.* Figure 2. **Lower panel:** The distribution assuming that the cosmopolitan species are governed by a truncated distribution, as in the lower panel of Figure 2. The data in Figure 3 upper panel agree with the middle panel, but not with the lower.

It is certainly consistent with a truncated exponential, where no site has less than ~40 species, and such a modification can also be handled with the methods of statistical mechanics. However, if there are no sites with fewer species than ~40, it would seem that there will be fewer pairs of sites with a small number of shared species than obtaining for an exponential probability that is not truncated. This is indeed the case; in Figure 3 (lower panel) we plot the number of pairs of sites as a function of the number of species in common, for a relative receptivity proportional to 4.8−lnR. This distribution is grossly different from that of the data shown in Figure 3 (upper panel), and also that modeled assuming relative probabilities proportional to 3.04−lnR, shown in Figure 3 (middle panel). The latter represents an exponential site probability distribution that is not truncated, that yields a distribution of the number of pairs consistent with an exponential as a function of the number of common species, and that agrees with the data. (The way in which the model distributions in Figure 2; Figure 3 were generated is relegated to a discussion in Appendix A.)

The conundrum presented by these data can be summarized rather simply. The distributions in the Figure 2 upper and lower panels agree, but do not agree with Figure 2, middle panel. However, the distributions in Figure 3’s upper and middle panels agree, but do not agree with Figure 3, lower panel.

In Figure 3 the data in the upper panel agree very well with the calculation in the middle panel, calculated from our model, and guaranteed to be drawn from an underlying exponential probability distribution. It is also the case that the coefficient in the exponent can be calculated directly from the distribution s(n); the value is 0.147 and the data in the upper panel are consistent with this exponential.

## 4. Disentangling the Conundrum

The distribution of *s*(*n*) with *n*, shown in Figure 1, is consistent with an exponential for *n* > 1. Extrapolating back to *n* = 1 gives 270 species at just one site, yet in the data there are 670. Thus 400 of these cannot be part of the exponential distribution characteristic of *cosmopolitans*. At the same time it should be remembered that only species for *n* > 1 appear in the distribution of pairs. This suggests that the distribution of site receptivity with rank should be divided into two classes: For cosmopolitans an exponential that is not truncated, with a receptivity tending to zero as $R\to R_{0}$ (where $R_{0}\sim 21)$, but for the excess of ~400 a different distribution, given at least approximately by the difference between the upper and middle panels of Figure 2. This difference is significantly greater than zero for lnR≳1.5, and grows with increasing rank. The excess species are characterized by occurring at one site only as a result of specialization or an inability to disperse, in contrast to the members of the class of cosmopolitans. We call these species *endemics*, and of course the sum of these endemics over all ranks must add up to 400 species. This could be achieved by letting all sites have the same receptivity (~20), but the difference is better represented by letting the sites of rank *R* have a receptivity approximately 2*R*, so that the site of rank 20 has a receptivity 40 for the endemics. High rank sites are of low species richness; this pattern corresponds to an endemic receptivity that is greatest for those sites poor in cosmopolitan species.

It should of course be noted that, with only 917 species in the data sample, the statistics are barely adequate to establish the division into the two classes of cosmopolitan and endemic species; there is no hope of finding finer structure. It is, however, intriguing that the distribution of receptivities for endemics is such that the overall distribution, endemics + cosmopolitans, is consistent with being drawn from a (truncated) exponential.

## 5. Discussion and Conclusions

Although it is not directly relevant, it may be worth pointing out that such inference of two classes of species overlapping is not unprecedented. In [9] a species abundance distribution for estuarine fish was decomposed into a sum of two popular forms; here we have examined, not a species abundance distribution, but several distributions characterizing the abundance of species.

The data of [7] make available the distribution of the number of species with the number of sites at which they are found, the number of species found at each site, and the number of pairs of sites as a function of the number of species shared. These distributions are consistent with cosmopolitan species (those capable of existing in more than one site) being distributed according to a statistical mechanics of division of some resource at random, and the number of species at sites of any given rank being drawn from an underlying exponential reflecting division among sites of that same resource. This is a second example of the structures first revealed, for alien species, in [5] and treated most completely in [6]. (Note: The reader familiar with [5] and [6] may recall that the population distribution over the sites is consistent with being drawn from an underlying exponential distribution that is not truncated. In those data all species are alien to all sites; there are no *endemics.*)

From the point of view of community assembly and population dynamics, the surprising feature of these patterns is the implication that the rate at which a given species loses or gains a site must be independent of the number already occupied. Similarly the rate at which a site gains or loses receptivity (in a dynamical interpretation) for cosmopolitan species must be independent of that receptivity. In the language of Maximum Entropy, these correspond to uniform *priors.*

Beyond that, there is evidence for a different dynamic operating in this environment. There are also *endemic* species, each incapable of flourishing outside of a single site. The distribution of the number of endemic species over sites is markedly different from the receptivities for the cosmopolitans. So far as one can extract anything further from these data, there seems to be an anti-correlation between receptivities for cosmopolitans and for the endemic species. Thus this study has further illuminated the application of statistical mechanics to population dynamics and community assembly, and has also suggested an ecologically interesting aspect of the interactions between endemic and cosmopolitan species, perhaps a form of biotic resistance.

## Appendix A

The underlying idea is that the distribution of the number of species with the number of sites at which they are found is an exponential generated by subdividing some underlying resource, and that the distribution of the number of sites as a function of their rank is drawn from an underlying exponential generated by subdividing that same resource along a different axis. Envisage a grid with species identifiers on one axis and site identifiers along the other. If a given species is found at a given site, then that intersection has value of 1. If a given species is not at that given site, that intersection has value zero. The sum of all elements represents the resource and was called the *footprint* in [6]. The grid must be populated according to some scheme that generates the exponential *s*(*n*) and the receptivities or rank abundance consistent with having been drawn from an underlying exponential distribution. Once the grid has been so populated the number of pairs of sites as a function of the number of species in common is easily generated by interrogating the grid.

There are various ways of populating the grid, listed in [6]. For the purposes of this note, we have populated the grid in the following way. First, for a given exponential distribution of *s*(*n*) the species are allocated numbers such that the first *s*(1) species are to be assigned to one site only, the next *s*(2) are to be assigned to two sites only, and so on. This step is trivial; less trivial is how the species are assigned to particular sites. The sites we label by rank and in assigning a given species to a site, the recipe is very simple. Each site of rank *R* has relative probability $lnR_{0}-lnR$ and a species is allocated to an empty site at random, according to the appropriately normalized probabilities. For the case of a pure exponential underlying the populations of 20 sites, $lnR_{0}$ has value 3.04 (*ln*21) and this value was used to generate Figure 2 and Figure 3, middle panels. The data we are discussing (Figure 2, upper panel) suggest a truncated exponential with $lnR_{0}$ ~4.8; hence Figure 2 and Figure 3, lower panels.

## References

[1] Pueyo S., He F., Zillio T. (2007). The maximum entropy formalism and the idiosyncratic theory of biodiversity. Ecol. Lett..

[2] Harte J. (2011). Maximum Entropy and Ecology.

[3] Bowler M.G., Kelly C.K. (2012). On the statistical mechanics of species abundance distributions. Theor. Popul. Biol..

[4] Bowler M.G. (2014). Species abundance distributions, statistical mechanics and the priors of maxent. Theor. Popul. Biol..

[5] Kelly C.K., Blundell S.J., Bowler M.G., Fox G.A., Harvey P.H., Lomas M.R., Ian Woodward F. (2011). The statistical mechanics of community assembly and species distribution. N. Phytol..

[6] Bowler M.G., Kelly C.K. (2017). On the statistical mechanics of alien species distribution. Entropy.

[7] Trejo I., Dirzo R. (2002). Floristic diversity of Mexican seasonally dry tropical forests. Biodivers. Conserv..

[8] MacArthur R.H. (1960). On the relative abundance of species. Am. Nat..

[9] Magurran A.E., Henderson P.A. (2003). Explaining the excess of rare species in abundance distributions. Nature.

